# Intraspecific variability modulates interspecific variability in animal organismal stoichiometry

**DOI:** 10.1002/ece3.981

**Published:** 2014-03-26

**Authors:** Rana W El-Sabaawi, Joseph Travis, Eugenia Zandonà, Peter B McIntyre, David N Reznick, Alexander Flecker

**Affiliations:** 1 Department of Ecology and Evolutionary Biology, Cornell UniversityIthaca, NewYork, 14853; 2 Department of Biological Science, Florida State UniversityTallahassee, Florida, 32306; 3 Department of Biology, Drexel UniversityPhiladelphia, Pennsylvania, 19104; 4 Center for Limnology, University of WisconsinMadison, Wisconsin, 53706-1413; 5 Department of Biology, University of CaliforniaRiverside, California, 92521

**Keywords:** Carbon, guppies, Hart's killifish, life history, nitrogen, phosphorus, predation, Trinidad

## Abstract

Interspecific differences in organismal stoichiometry (OS) have been documented in a wide range of animal taxa and are of significant interest for understanding evolutionary patterns in OS. In contrast, intraspecific variation in animal OS has generally been treated as analytical noise or random variation, even though available data suggest intraspecific variability in OS is widespread. Here, we assess how intraspecific variation in OS affects inferences about interspecific OS differences using two co-occurring Neotropical fishes: *Poecilia reticulata* and *Rivulus hartii*. A wide range of OS has been observed within both species and has been attributed to environmental differences among stream systems. We assess the contributions of species identity, stream system, and the interactions between stream and species to variability in N:P, C:P, and C:N. Because predation pressure can impact the foraging ecology and life-history traits of fishes, we compare predictors of OS between communities that include predators, and communities where predators are absent. We find that species identity is the strongest predictor of N:P, while stream or the interaction of stream and species contribute more to the overall variation in C:P and C:N. Interspecific differences in N:P, C:P, and C:N are therefore not consistent among streams. The relative contribution of stream or species to OS qualitatively changes between the two predation communities, but these differences do not have appreciable effects in interspecific patterns. We conclude that although species identity is a significant predictor of OS, intraspecific OS is sometimes sufficient to overwhelm or obfuscate interspecific differences in OS.

## Introduction

Organismal stoichiometry (OS) defined as the ratios of elements in animals is an important trait because it is used to calculate nutritional demand, and because of its potential to constrain a range of ecological processes (Elser and Urabe 1999; Elser et al. 2000; Frost et al. 2006). Mismatches between elemental requirements of animals and the elemental content of their diets have significant consequences for many ecological and biogeochemical processes, including population dynamics, feeding rates, nutrient recycling, and competition (Elser and Urabe 1999; Anderson et al. 2004; Hessen et al. 2004; Fink and Von Elert 2006; McManamay et al. 2010). Characterizing interspecific patterns in OS can also improve our understanding of the links between evolutionary innovations and biogeochemical cycling, and can clarify evolutionary patterns in nutritional requirements and thresholds (Kay et al. 2005; Frost et al. 2006).

Among species, OS varies with body size, morphology, and life-history traits because these traits affect biochemical composition, which ultimately controls the elemental composition of animals (Tanner et al. 2000; Sterner and Elser 2002; Gonzalez et al. 2011). There can also be significant OS differences among trophic guilds presumably because diet constrains elemental availability (Fagan et al. 2002; Frost et al. 2006; McIntyre and Flecker 2010). For example, animals that feed on high-quality carnivorous diets have been reported to have higher %N or %P and lower %C than animals that feed on low-quality herbivorous or detrital diets (Fagan et al. 2002; McIntyre and Flecker 2010).

Within these trophic guilds, animals are assumed to be homeostatic, meaning that they regulate their elemental composition through homeostatic mechanisms, thereby buffering themselves from variability in the elemental composition of their diets (Persson et al. 2010). This regulation is thought to dampen intraspecific OS variability in animals (Karimi and Folt 2006). However, recent studies have shown that OS can still vary dramatically within species due to intraspecific variability in trait distributions (Pilati and Vanni 2007; Gonzalez et al. 2011), or to variability in abiotic factors such as temperature, nutrients, and light (Dickman et al. 2008; Hamback et al. 2009; Small and Pringle 2010). Environmental factors influence animal OS either by acting directly on organismal traits (Hamback et al. 2009), or by altering the availability of elements for consumers by altering elemental content of their resources (Schade et al. 2003; Small and Pringle 2010). Some of this intraspecific variability is due a relaxation of homeostasis (Small and Pringle 2010), but a recent compilation showed that the majority of animals are largely homeostatic (Persson et al. 2010). The consequences of intraspecific OS variability in animals are poorly understood, but are likely to be important (Nakazawa 2011).

A striking aspect of studies on intraspecific stoichiometry is that they commonly report ranges of elemental content within a single species that are comparable to ranges of elemental content observed across almost all species of the same taxa (Pilati and Vanni 2007; Bertram et al. 2008; El-Sabaawi et al. 2012b). The implication of these studies is that exogenous or endogenous factors that influence OS within a species might also influence the magnitude and, in some cases, perhaps the direction of stoichiometric variation between species. For example, nutrient enrichment affects the OS of some, but not all resident invertebrates in a P-limited stream, suggesting that interspecific OS variability in that community depends on intraspecific differences in the response to P availability (Cross et al. 2003). However, few studies examine inter- and intraspecific OS variability simultaneously, so it is unclear if intraspecific variability has a qualitative effect on the pattern of interspecific variation.

In this study, we test whether intraspecific variability in elemental composition affects interspecific patterns in stoichiometry using two species of coexisting freshwater fish. On the island of Trinidad, *Poecilia reticulata* (the guppy) and *Rivulus hartii* (Hart's killifish) coexist across a range of streams that vary in environmental conditions (Kohler 2010). Each species displays a wide range of intraspecific variability in OS that is comparable with the range of OS values reported across all freshwater fish species (e.g., P content between 1 and 5% of dry mass) (El-Sabaawi et al. 2012a,b). The OS of both species varies significantly among different streams. The cause of this spatial OS variability has not been specifically identified but appears to be broadly related to stream-specific differences in nutrient cycling. For example, in *P. reticulata*, body N is significantly correlated with dissolved N concentrations, and P content appears to be influenced by the presence of limestone deposits, which commonly reduce dissolved P concentrations and P content in basal resources in aquatic systems (Wetzel 2001; El-Sabaawi et al. 2012b). In *R. hartii*, OS variability is significantly correlated with the stoichiometry of basal resources (i.e., benthic organic matter and epilithon), which is correlated with the availability of dissolved nutrients (El-Sabaawi et al. 2012a; Kohler et al. 2012).

Within each stream, *P. reticulata* and *R. hartii* also exist in different types of fish communities whose composition is determined by waterfalls that restrict the upstream movement of large predatory fish (Magurran 2005). In downstream sites, located below the first waterfall barrier in the stream, *P. reticulata* and *R. hartii* are found with a variety of fish predators, but above these barriers, the community is limited to *P. reticulata* and *R. hartii* (Gilliam et al. 1993). The presence of predators acts as a selective agent on the life-history traits and other phenotypic characteristics of both species (Reznick et al. 1990; Walsh and Reznick 2011). In sites where *P. reticulata* and *R. hartii* co-exist with predators (high predation, HP), both species grow quickly and produce abundant, small offspring (Reznick and Endler 1982; Walsh and Reznick 2009). In sites where *P. reticulata* and *R. hartii* are the only fish taxa (Low predation, LP), both species grow more slowly and produce fewer, larger offspring (Reznick and Endler 1982; Walsh and Reznick 2009). In LP communities, the %C, C:N, and C:P of female *P. reticulata* and adult *R. hartii* are slightly but significantly elevated compared with other types of communities. However, OS differences caused by differences in predation pressure are small relative to the spatial (i.e., stream) differences in OS (El-Sabaawi et al. 2012a,2b).

Although the presence of predators does not appear to be a strong predictor of OS in either *P. reticulata* or *R. hartii*, it might constrain their diets, thereby affecting their ability to acquire elements. *P. reticulata* consume higher proportions of low-quality algae and detritus in LP sites than they do in HP sites, where their diet is primarily composed of high-quality invertebrates (Zandona et al. 2011). On the other hand, *R. hartii* diets in HP and LP sites are composed largely of aquatic invertebrates and do not appear to be affected by predators (Fraser et al. 1995). These patterns imply that the diets of both species, and the ultimate constraint on their ability to acquire elements, are more similar in HP than they are in LP communities. If this is true, then the effect of intraspecific OS on interspecific OS differences might be stronger in LP compared to HP communities.

Here, we use a recently published dataset (El-Sabaawi et al. 2012a,b) to assess how intraspecific OS variability affects observed interspecific OS patterns. Our overarching question is: how robust are interspecific patterns in OS when the OS of individual species also varies? To answer the question, we compare the relative effect sizes of inter- and intraspecific predictors of OS. We analyze each stoichiometric ratio using a statistical model that includes species identity, stream identity and their interaction. Species identity represents interspecific differences, while stream identity is the primary predictor of intraspecific OS variability within each species. A significant interaction term suggests that interspecific differences in OS are not consistent among streams, or that intraspecific variability in OS is large enough to affect interspecific OS differences. We also test whether the relative contributions of stream and species vary between predation regimes by including predation community (LP or HP), and the interaction of predation with species and stream, in the model. Significant interactions between predation and these terms would suggest that predation alters the relative influence of intra- and interspecific predictors of OS. Because *P. reticulata* and *R. hartii* likely use similar resources in HP environments but different resources LP environments, we predict that their responses to stream conditions would be more similar in HP compared with LP communities. This would make interspecific OS more robust in HP compared with LP communities and would result in the OS of guppies and *R. hartii* being more strongly correlated with each other in HP than in LP communities.

## Materials and methods

Our study is a new synthesis of two recently published datasets that have documented the causes of intraspecific variability of OS in each species (El-Sabaawi et al. 2012a,b). These papers examined each species separately. However, because both species were sampled from exactly the same sites, it is possible to merge the datasets to assess the relative influence of interspecific OS variability on intraspecific OS patterns. The sampling scheme and analysis were described thoroughly in the previous papers. Briefly: *R. hartii* and *P. reticulata* were sampled from six streams in Trinidad: Arima, Aripo, Guanapo, Quare, Turure, and the Marianne River. In each stream, fish were sampled from an HP and an LP location separated by a waterfall, yielding a total of 12 sampled populations per species. Classification of predation community was confirmed by the presence or absence of fish predators such as the pike cichlid (*Crenicichla* sp), or the wolffish (*Hoplias malabaricus*) (Strauss 1990). A total of ∼ 400 fish were analyzed for stoichiometry, including 230 *P. reticulata* and 170 *R. hartii*. Average total number of fish sampled from each site was ∼ 33 and ranged from 17 (Aripo HP) to 50 (Guanapo LP). *P. reticulata* are relatively small (max. length ∼35–40 mm) live-bearing fish, whereas *R. hartii* are larger (max. length ∼ 100 mm), egg-bearing fish.

Guts and reproductive tissues (including eggs) were removed prior to elemental analysis. Gutting fish is necessary for stoichiometric analysis because guts vary in fullness among and within species. We felt it was necessary to remove reproductive tissues completely because some of these tissues were inevitably removed while the fish were being gutted, and our goal was to standardize any potential biases across all fish. We do not believe that this would cause a significant bias in our data because reproductive tissues are typically a very small component of the body (El-Sabaawi et al. 2012b). Fish were oven-dried (55°C, 7 days or until constant weight achieved) and ground into a fine powder using a mortar and pestle. Subsamples (∼5 mg) were analyzed for %C and %N using a Carlo Erba NA1500 CHN analyzer. Subsamples (∼ 1 mg) for %P analysis were first ashed at 500°C for 1 h and then digested with HCl at 102°C for 2 h. The concentration of dissolved P in the digested solution was measured using the molybdate-blue method (Parsons et al. 1984). Bone meal (NIST #1486) was used as an internal standard, and the efficiency of P extraction was typically >95%. Triplicate subsamples were analyzed whenever possible.

Organismal stoichiometry (OS) was modeled using a series of general linear models for each stoichiometric ratio. The first series of models, referred to as “global” models, included Stream (6 levels), Species (2 levels), Predation (2 levels), body size and interactions between the main effects. In order to further dissect the influence of predation on OS, we then separated the data by predation community and ran a general linear model containing stream, species, body size and their interaction for each stoichiometric ratio. We did not distinguish between males, females or juveniles because sex and stage of development were found to be relatively weak predictors of OS in both species (El-Sabaawi et al. 2012a,b). Although body size was also a weak predictor of OS in these species, the strength of the correlation between body size and OS could vary among species (Dantas and Attayde 2007). Body size and its interaction with species were therefore included as predictors in each predation-specific model but were retained only when they were statistically significant (*P* < 0.05) and when removing them led to a significant changes in the corrected Akaike Information Criterion (AICc).

These analyses were performed on stoichiometric ratios (i.e., N:P, C:P, and C:N), which were log-transformed in order to meet assumptions for normality and homogeneity of variance. We also ran the global models on individual elements (%C, %N, and %P) to clarify patterns observed in the stoichiometric ratios. Effect size was estimated using partial eta squared (*η*^2^), defined as the sum of squares of the individual factor divided by the total of the sum of squares of the factor added to the sum of squares of the error (Petraitis 1998). A larger value of this metric indicated that a predictor explained a larger portion of the variance compared to a smaller value.

## Results

### General patterns

Compared with *R. hartii P. reticulata* had higher average %P (∼3.7% vs. ∼3.1%), lower average %N (∼9.3% vs. ∼10.7%), and lower average %C (∼40.8% vs. ∼41.7%) (Table 1). Both species were nearly equally variable in terms of %P (CoV ∼20%), but *P. reticulata* had higher coefficients of variability than *R. hartii* for %N (11% vs. 8%) and %C (9% vs. 7%). Both species had more variable (i.e., higher CoVs) stoichiometry and elemental composition in LP compared to HP communities in %P, %N, %C, N:P, and C:N but not C:P (Table 1).

**Table 1:** Summary statistics (means, standard deviations [Std Dev], and coefficients of variability [CoV]) of *Poecilia reticulata* and *Rivulus hartii* from LP and HP communities

Variable	Stastic	*Poecilia reticulata* HP	*Rivulus hartii* HP	*Poecilia reticulata* LP	*Rivulus hartii* LP
%P	Mean	3.7	3.2	3.6	3.1
	Std Dev	0.7	0.6	0.8	0.7
	CoV	19.0	19.3	21.0	20.9
%N	Mean	9.5	10.6	9.4	10.8
	Std Dev	0.9	0.7	1.2	1.0
	CoV	9.1	6.8	12.4	9.0
%C	Mean	40.1	41.2	41.4	41.8
	Std Dev	2.8	2.5	4.4	3.9
	CoV	7.0	6.2	10.5	9.2
N:P	Mean	6.0	7.6	6.0	8.0
	Std Dev	1.3	1.8	1.7	1.9
	CoV	21.6	23.9	28.8	24.1
C:P	Mean	29.5	34.6	31.1	36.3
	Std Dev	7.1	9.4	8.5	10.0
	CoV	23.9	27.1	27.5	27.6
C:N	Mean	5.0	4.5	5.2	4.5
	Std Dev	0.6	0.3	0.7	0.5
	CoV	11.3	7.1	13.8	10.9

The global models for N:P, C:P, and C:N showed that species differences in stoichiometric ratios were always significant (i.e., the species term was a significant predictor of all ratios), but they did not always vary the same way among streams (i.e., stream × species interactions were also significant predictors of all ratios). This meant that intraspecific OS (caused by differences among streams) was large enough to alter observed interspecific differences in all three stoichiometric ratios. Although its effects were weak, body size was a significant predictor of N:P, C:P, but not C:N (Table 2). A significant size × species interaction indicated that the slope of the relationship between body size and C:P and N:P varied slightly between the species. Predation was not a significant predictor of stoichiometry, but the interaction between predation × stream × species were always weakly significant, suggesting that predation altered how fish OS responded to local conditions.

**Table 2:** Global models for N:P, C:P, and C:N. All elemental ratios were log transformed prior to analysis. Rank is the rank order of each variable based on partial *η*^2^. The most important explanatory variables are bolded. The species effect represents interspecific differences, while the stream effect represents intraspecific differences. The interaction of stream × species indicates that interspecific patterns vary among different streams, and indicate that intraspecific variability alters observed differences among species

Variables	F Ratio	P value	Partial *η*^2^	Rank of effect
A. Model of N:P				
Stream	3.664	0.003	0.047	4
**Species**	**83.772**	**<0.0001**	**0.183**	**1**
Size	8.950	0.003	0.023	6
Predation	0.643	0.4232	0.002	9
Predation*Stream	4.985	0.0002	0.062	3
Size*Species	4.650	0.0317	0.012	7
Stream*Species	14.008	<0.0001	0.158	2
Predation*Species	0.656	0.4187	0.002	8
Predation*Stream*Species	2.294	0.045	0.030	5
Error				
B. Model for C:P				
Stream	1.738	0.1249	0.023	5
Species	30.778	<0.0001	0.076	3
Size	8.386	0.004	0.022	6
Predation	1.845	0.1752	0.005	8
Predation*Stream	6.410	<0.0001	0.079	2
Size*Species	4.220	0.0406	0.011	7
Stream*Species	**8.897**	**<0.0001**	**0.106**	**1**
Predation*Species	0.001	0.9747	0.000	9
Predation*Stream*Species	2.291	0.0453	0.030	4
Error				
C. Model for C:N				
**Stream**	**39.923**	**<0.0001**	**0.348**	**1**
Species	57.600	<0.0001	0.133	3
Size	0.396	0.5294	0.001	8
Predation	2.731	0.0993	0.007	7
Predation*Stream	12.126	<0.0001	0.140	2
Size*Species	0.141	0.7079	0.000	9
Stream*Species	8.384	<0.0001	0.101	4
Predation*Species	3.306	0.0698	0.009	6
Predation*Stream*Species	3.951	0.0017	0.050	5
Error				

Running these global models on individual elements (%P, %N, and %C) revealed slightly different trends, but the general conclusions are similar to those drawn from the global analysis of elemental ratios (Appendix S2, Appendix S3). Species differences were significant for %P and %N, but not in %C. Stream differences were significant in %N and %C, but not %P. Stream × species interactions were significant predictors of all of the elements, suggesting that interspecific differences in elemental content were not consistent among streams. Similarly, the interaction between predation × stream × species were significant (or marginally significant in terms of %C), suggesting that the relative influence of interspecific and intraspecific variability on elemental composition varied between predation communities. Body size has a significant effect on %P, but not %N and %C, which is consistent with predictions based on other fish studies. Predation had a significant effect on %C because fish from LP communities had slightly more %C (41.6% compared with 40) than fish from HP communities. This effect was similar between species as the interaction of predation × species was not significant. However, a significant predation × stream interaction indicated that the effect of predation on%C was not consistent among streams. A general conclusion from all global models shows that although there are detectable interspecific differences in elemental composition and stoichiometry between *R. hartii* and *P. reticulata*, these differences are strongly mediated by local stream conditions, and occasionally by the type of community the fish are found in. In the next section, we explore these patterns further by looking at the influence of stream identify and species identify within each predation community.

### Predation-specific models

#### The role of body size in the predation-specific models

Although the effects of body size on OS were generally small in the predation-specific models, we retained them because their removal significantly altered model fit (as indicated by AICc, data not shown). In general, body size was a stronger predictor of OS in LP compared with HP communities, but there were also differences in the body size effect among elemental ratios (Table 3).

**Table 3:** Results of a general linear model (GLM) analysis on stoichiometric ratios in *P. reticulata* and *Rivulus hartii* from high-predation (HP) and low-predation (LP) communities. Values are F-ratios except where indicated. In all GLMs model, degrees of freedom were between 11 and 15, while error degrees of freedom were between 163 and 165 for HP models, and between 189 and 191 for LP models. “R” indicates that effect was removed because it was not statistically significant and because it did not contribute to model fit (See text for detail)

Ratio	Variables	HP	LP
N:P	Stream	0.74	6.1**
	Species	29.5**	65.0**
	Stream × Species	2.9*	12.5**
	Size	3.4	8.2**
	Size × Species	8.9*	R
	*r*^2^	0.3**	0.52**
C:P	Stream	3.8*	3.5*
	Species	13.3**	19.3**
	Stream × Species	2.6*	7.9**
	Size	3.9*	6.5*
	Size × Species	7.5*	R
	*r*^2^	0.34**	0.33**
C:N	Stream	33.6**	21.7**
	Species	84.9**	110.7**
	Stream × Species	4.0**	6.4**
	Size	R	R
	Size × Species	R	R
	*r*^2^	0.62**	0.55**
N:P	Stream	0.74	6.1**
	Species	29.5**	65.0**
	Stream × Species	2.9*	12.5**
	Size	3.4	8.2**
	Size × Species	8.9*	R
	*r*^2^	0.3**	0.52**
C:P	Stream	3.8*	3.5*
	Species	13.3**	19.3**
	Stream × Species	2.6*	7.9**
	Size	3.9*	6.5*
	Size × Species	7.5*	R
	*r*^2^	0.34**	0.33**
C:N	Stream	33.6**	21.7**
	Species	84.9**	110.7**
	Stream × Species	4.0**	6.4**
	Size	R	R
	Size × Species	R	R
	*r*^2^	0.62**	0.55**

* *P* values < 0.05.

** *P* values < 0.001.

In HP, communities body size was not a significant predictor of N:P, but it had a significant interaction with species (Table 3). In contrast, body size was negatively and significantly correlated with C:P, while also having a significant interaction with species (Table 3). The interactions were significant because body size was more strongly (negatively) correlated with N:P and C:P in *P. reticulata* (*r*^2^ = 0.06, *F*_1,103_ = 7.3, *P* = 0.008 for N:P, *r*^2^ = 0.05, *F*_1,103_=5.3, *P* = 0.015 for C:P) than in *R. hartii* (*P* > 0.05). In LP communities, body size was a significant predictor of N:P and C:P (Table 1), but its interaction with species was not significant (Table 3, Table 4). Body size and its interaction with species were not significant predictors of C:N in either community (Table 3, Table 4).

**Table 4:** Effect sizes of the predation-specific models reported in Table 3 measured as partial eta squared (partial *η*^2^). Larger values of partial *η*^2^ indicate that the variable predicts a larger portion of the variance. HP refers to high-predation communities. LP refers to low-predation communities

Ratio	Variables	HP	LP
N:P	Stream	0.02	0.14
	Species	0.15	0.25
	Stream × Species	0.08	0.25
C:P	Stream	0.10	0.08
	Species	0.07	0.09
	Stream × Species	0.07	0.17
C:N	Stream	0.50	0.36
	Species	0.34	0.36
	Stream × Species	0.11	0.14

#### General trends in the predation-specific models

**Figure 1:**
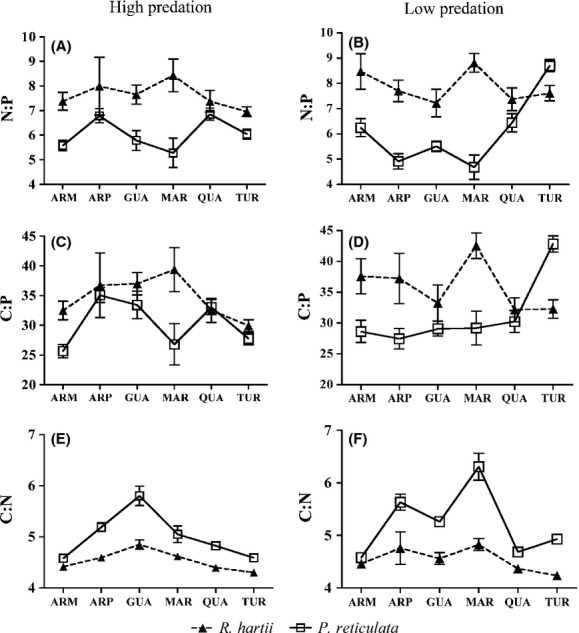
Averages (and standard errors) of organismal stoichiometry (N:P, C:P, and C:N) of *P. reticulata* and *R. hartii* collected from each stream, and from of the two predation communities.

Species, stream and their interaction were all significant predictors of OS (Table 2, Table 3, Fig. 1), but the importance of each predictor varied among elemental ratios, and between predation communities. Species was the strongest predictor of N:P, while stream or stream × species were the strongest predictors of C:N and C:P, respectively. The significant stream × species interactions in all of the ratios confirmed that species differences varied (i.e., were not consistent) among streams. In general, *P. reticulata* had lower N:P, C:P ratios and higher C:N ratios than *R. hartii*, but interspecific differences were large in some streams, and small in others. In the Turure LP site species, differences in n:p and c:p were the opposite of those observed in other streams, with *P. reticulata* having substantially higher levels of n:p and c:p than *R. hartii* (Fig. 1).

#### Variability in N:P

Species explained most of the variability of n:p in both predation communities (Table 1 and 4). The N:P of *R. hartii* was higher than *P. reticulata* N:P in 11 of the 12 populations (Fig. 1a,b). Although it was significant in both communities, the interaction of stream × species was a stronger predictor of N:P in LP compared with HP communities (Table 4). This pattern was driven primarily by the Turure LP site, where *P. reticulata* had significantly higher N:P than *R. hartii* (Fig. 1b).

#### Variability in C:P

Species, stream and the stream × species were significant predictors of C:P, but the relative importance of these factors differed between predation communities (Tables 3 and 4). Stream was the strongest contributor to C:P in fish from HP communities (Table 4). Differences in C:P among streams were stronger than differences in C:P between the two species. The significant interaction of stream × species was driven by the Marianne, where interspecific differences in C:P were much bigger than they were in other streams (Fig. 1c). The stream × species interaction was the strongest predictor of C:P in fish from LP sites (Tables 3 and 4). In most LP sites, *R. hartii* C:P was higher than *P. reticulata* C:P, but differences between species varied widely among streams (Fig. 1d). As was the case for N:P, interspecific patterns in the C:P from the Turure were the opposite of the pattern observed in other streams (Fig. 1d).

#### Variability in C:N

*Poecilia reticulata* C:N was higher than *R. hartii* C:N in almost all locations (Fig. 1e,f), but differences in C:N among streams were bigger than the difference in C:N between the two species (Fig. 1e,f). The interaction of stream species was an important contributor to C:N, especially in fish from LP communities (Table 4, Fig. 1f).

#### Differences between predation communities

The stream effect was a more important predictor of OS in HP compared with LP communities, although its effect was relatively small in the N:P model. The species term was a weaker predictor of OS in HP compared with LP communities, although its effect was relatively small in the C:P model. The stream × species was a stronger predictor of OS in LP compared with HP communities.

**Figure 2:**
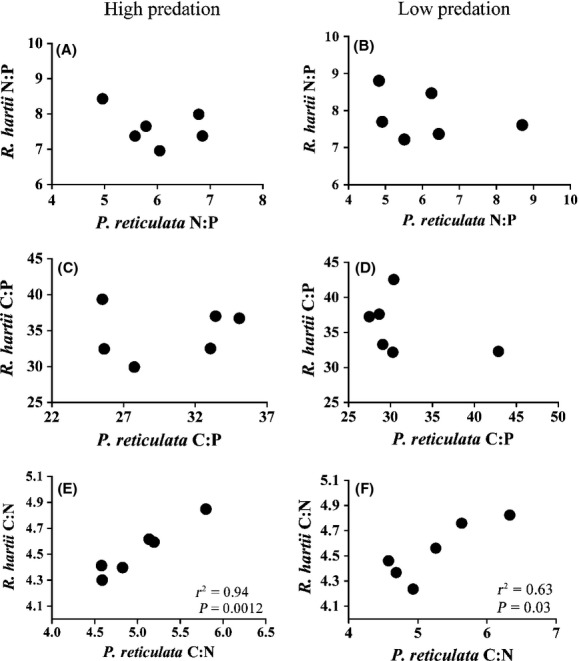
Correlations between average *P. reticulata* and average *R. hartii* stoichiometry in high-predation and low-predation communities.

Despite qualitative differences between the predation communities, it was unclear whether predation led to appreciable, observable differences in interspecific OS. For example, a stronger stream effect in the OS of fish from HP communities did not lead to stronger correlations between *P. reticulata* and *R. hartii* stoichiometry in HP compared with LP communities. *P. reticulata* N:P (or C:P) and *R. hartii* N:P (or C:P) were not significantly correlated in either HP or LP communities (Fig. 2a–d). However, *P. reticulata* C:N and *R. hartii* C:N were significantly correlated in both predation communities, and this correlation was considerably stronger in HP compared with LP communities (*r*^2^ = 0.94 in HP sites, and *r*^2^ = 0.64 in LP sites) (Fig. 2e,f).

## Discussion

Currently, ecological stoichiometry studies tend to emphasize interspecific differences in animal OS, while considering intraspecific differences to be relatively small and inconsequential. Yet more and more studies are reporting considerable intraspecific stoichiometry in many taxa of terrestrial and aquatic consumers (Hendrixson et al. 2007; Bertram et al. 2008). In this study, we tested whether intraspecific variability in OS within two species of coexisting fish had the potential to affect observed stoichiometric differences between the two fish species. We showed that intraspecific variability in OS could alter the magnitude and direction of interspecific differences in elemental composition (Fig. 1). We reported interspecific differences in OS between *P. reticulata* and *R. hartii* in a number of streams, but these patterns varied considerably depending on the stream from which fish were sampled. This suggested that factors that influence intraspecific animal OS, which in this case were related to environmental and biogeochemical differences among streams, could also alter interspecific differences in OS, at least between species occupying similar trophic positions like *P. reticulata* and *R. hartii*.

Although there are several studies on the elemental composition of fish, very few relate elemental composition to biochemical composition, and little is known about the relative sensitivity of elemental composition to environmental conditions. The majority of %P in fish is determined by bone content, but up to a third of P in fish is bound to more labile biochemical pools such as ATP, nucleic acids or phospholipids (Sterner and Elser 2002; Hendrixson et al. 2007; Pilati and Vanni 2007). However, bone acts as a reservoir of P and calcium in fish, and a fraction of bone can be resorbed when either of these elements become limiting (Witten and Huysseune 2009). Nitrogen reflects protein concentrations, but a portion of N is also found in other molecules such as nucleic acids (Gnaiger and Bitterlich 1984). Carbon is assumed to vary primarily with lipid content and carbohydrates, both of which are sensitive to food availability and to reproductive state (Gnaiger and Bitterlich 1984). Based on these studies, we would expect fish C to be more labile than either N or P. In agreement with this expectation, we find that species differences are strongest in %P and %N, although both elements are also sensitive to environmental conditions among streams (as suggested by a significant stream or stream × species interaction) (Appendix S2,S3). Percent C is most strongly related to stream (i.e., environmental conditions), and to predation, which selects for life-history trade-offs in both species (Appendix S2).

Predation has a significant effect on population dynamics, community interactions, and trophic ecology. Fear of predators might also alter OS by increasing metabolic rates and decreasing foraging (Hawlena and Schmitz 2010). In the *P. reticulata* -*R. hartii* system, predation affects phenotypic traits of both species, and the foraging ecology of *P. reticulata* (Zandona et al. 2011). Previously, we have shown that predation is a weak yet significant predictor of OS in both *P. reticulata* and *R. hartii* and that the effect of predation on OS was much weaker than the effects of streams (El-Sabaawi et al. 2012a,b). The findings are confirmed in the global models (Table 2), which suggest that the presence or absence of predators affects how interspecific OS responds to stream variability. In the predation-specific models, the interaction of stream × species contributes more to OS variance in LP than HP communities, and this observation provides support for our predictions that species differences in OS might be more sensitive to intraspecific variability in LP than in HP communities. However, contrary to our prediction, the OS of both species are not more strongly correlated in HP communities compared with LP communities, nor are the observations consistent for all stoichiometric ratios we considered (Fig. 2). We conclude that although there are qualitative differences in the relative contributions of predictors of OS in fish from HP versus LP communities, these differences do not have an appreciable effect on observable patterns in interspecific OS.

Differences in the stream × species interaction between HP and LP sites might also arise from differences in ambient environmental heterogeneity within each predation community. The OS of both species is more variable in LP than in HP communities (Table 1). The higher contribution of the stream × species interaction to N:P and C:P in LP sites might simply reflect that this subset of sites was more heterogeneous in environmental/dietary P availability than the corresponding subset of HP sites.

Significant stream × species interactions might also arise from interspecific or phenotypic differences in homeostatic regulation. Variability in the strength of elemental homeostasis modulates the response of invertebrate taxa to stream conditions (Small and Pringle 2010). Although recent syntheses of the strength of homeostasis across a large number of aquatic consumers have shown that fish can be considered strictly homeostatic (Persson et al. 2010), differences in elemental homeostasis among co-existing species or between phenotypes have not been directly assessed. We do not know whether there are differences in the strength of elemental homeostasis between *P. reticulata* and *R. hartii,* or whether adaptation to predators alters the strength of elemental homeostasis between phenotypes of the same species. A detailed comparative study of the strength of elemental homeostasis between predation-adapted phenotypes will likely be illuminating.

One potential bias in our study is that interspecific differences in OS between *P. reticulata* and *R. hartii* are relatively small compared with those observed across a wider number of fish taxa (McIntyre and Flecker 2010). However, it is important to note that intraspecific OS variability in *P. reticulata* and *R. hartii* is not unusually large compared with other fish taxa. Recently, similar ranges (and CoVs) of C, N, and P were reported in other fish species including gizzard shad (Pilati and Vanni 2007), bluegills (Hendrixson et al. 2007), and European perch (Vrede et al. 2011). Variable intraspecific OS is also reported in a large number of invertebrate taxa (Bertram et al. 2008). It is therefore likely that intraspecific OS variability is the norm rather than the exception, and that it can influence interspecific OS differences in many other taxa and systems. Describing and understanding interspecific variability in OS is important for a number of ecological and evolutionary questions (Vanni et al. 2002; Kay et al. 2005; Frost et al. 2006). However, our study demonstrates that characterizations of interspecific OS cannot assume that intraspecific differences within taxa are unimportant and must also account for the factors that create intraspecific variation in OS.

## Supporting Information

**Appendix S1.** Means of elemental composition (standard deviation) from each site.

**Appendix S2.** Global statistical models for individual elements.

**Appendix S3.** Averages (and standard errors) of elemental composition of *P. reticulata* and *R. hartii* collected from each stream, and from of the two predation communities.
