# Supplementary material for: Intraspecific variability modulates interspecific variability in animal organismal stoichiometry
Source: Ecol Evol. 2014 Mar 26;4(9):1505–15. doi: 10.1002/ece3.981 (PMC4063454; doi:10.1002/ece3.981)
Supplement: Supplementary file 1 [file ece30004-1505-SD1.docx]

Appendix 1. Means of elemental composition (standard deviation) from each site.

| Predation | River | *P. reticulata* %P | *R. hartii*  %P | *P. reticulata* %N | *R. hartii*  %N | *P. reticulata* %C | *R. hartii*  %C |
| --- | --- | --- | --- | --- | --- | --- | --- |
| HP | Arima | 4.0(0.6) | 3.3(0.6) | 9.8(0.5) | 10.6(0.8) | 38.4(2.8) | 40.1(2.3) |
| HP | Aripo | 3.2(0.4) | 3.05(0.6) | 9.65(0.7) | 10.7(1.0) | 42.8(1.6) | 42.0(3.9) |
| HP | Guanapo | 3.4(0.9) | 3.1(0.6) | 8.3(0.9) | 10.4(0.9) | 41.1(2.7) | 42.9(2.7) |
| HP | Marianne | 4.1(0.8) | 2.95(0.8) | 8.7(0.5) | 10.4(0.6) | 38.3(3.2) | 41.2(2.9) |
| HP | Quare | 3.2(0.4) | 3.4(0.7) | 9.9(0.5) | 10.8(0.5) | 40.8(1.5) | 40.6(1.7) |
| HP | Turure | 3.8(0.6) | 3.6(0.3) | 10.2(0.5) | 11.1(0.5) | 40.2(2.0) | 40.9(1.6) |
| LP | Arima | 3.7(0.7) | 3.0(0.5) | 10.0(0.5) | 10.85(0.5) | 39.4(2.4) | 41.4(1.6) |
| LP | Aripo | 4.1(0.7) | 3.4(0.9) | 8.7(0.9) | 11.3(1.4) | 41.8(2.3) | 45.1(3.4) |
| LP | Guanapo | 3.8(0.7) | 3.3(0.8) | 9.1(1.2) | 10.2(0.9) | 41.4(6.0) | 40.0(4.1) |
| LP | Marianne | 3.8(0.7) | 2.8(0.6) | 8.1(1.1) | 10.6(0.7) | 43.4(3.0) | 43.8(3.3) |
| LP | Quare | 3.55(0.7) | 3.3(0.6) | 9.9(0.5) | 10.7(1.4) | 39.7(2.7) | 40.1(5.4) |
| LP | Turure | 2.6(0.3) | 3.3(0.5) | 10.3(0.5) | 11.3(0.7) | 43.5(2.5) | 40.9(1.9) |
|  |  |  |  |  |  |  |  |
