# Supplementary material for: Intraspecific variability modulates interspecific variability in animal organismal stoichiometry
Source: Ecol Evol. 2014 Mar 26;4(9):1505–15. doi: 10.1002/ece3.981 (PMC4063454; doi:10.1002/ece3.981)
Supplement: Supplementary file 2 [file ece30004-1505-SD2.docx]

Appendix 2: Global statistical models for individual elements. Rank is the rank order of each variable based on partial η^2^. The most important explanatory variables are bolded.

| a) Model for %P |  |  |  |  |  |  |  |
| --- | --- | --- | --- | --- | --- | --- | --- |
| Variable | Nparm | DF | Sum of Squares | F Ratio | P value | Partial η2 | Rank of effect |
| Stream | 5.000 | 5.000 | 1.121 | 0.585 | 0.712 | 0.008 | 7 |
| Species | 1.000 | 1.000 | 16.914 | 44.125 | <.0001 | 0.106 | 2 |
| Size | 1.000 | 1.000 | 5.537 | 14.445 | 0.000 | 0.037 | 4 |
| Predation | 1.000 | 1.000 | 0.134 | 0.349 | 0.555 | 0.001 | 8 |
| Predation*Stream | 5.000 | 5.000 | 11.425 | 5.961 | <.0001 | 0.074 | 3 |
| Size*Species | 1.000 | 1.000 | 2.357 | 6.147 | 0.014 | 0.016 | 6 |
| Stream*Species | **5.000** | **5.000** | **17.986** | **9.384** | **<.0001** | **0.112** | **1** |
| Predation*Species | 1.000 | 1.000 | 0.116 | 0.304 | 0.582 | 0.001 | 9 |
| Predation*Stream*Species | 5.000 | 5.000 | 4.112 | 2.145 | 0.045 | 0.028 | 5 |
| Error |  |  | 143.300 |  |  |  |  |
|  |  |  |  |  |  |  |  |
| b) Model for %N |  |  |  |  |  |  |  |
| Variable | Nparm | DF | Sum of Squares | F Ratio | P value | Partial η2 | Rank of effect |
| **Stream** | **5.000** | **5.000** | **79.649** | **23.662** | **<.0001** | **0.240** | **1** |
| Species | 1.000 | 1.000 | 44.757 | 66.483 | <.0001 | 0.151 | 2 |
| Size | 1.000 | 1.000 | 0.344 | 0.511 | 0.475 | 0.001 | 7 |
| Predation | 1.000 | 1.000 | 0.136 | 0.202 | 0.654 | 0.001 | 8 |
| Predation*Stream | 5.000 | 5.000 | 3.368 | 1.001 | 0.417 | 0.013 | 5 |
| Size*Species | 1.000 | 1.000 | 0.018 | 0.027 | 0.870 | 0.000 | 9 |
| Stream*Species | 5.000 | 5.000 | 20.594 | 6.118 | <.0001 | 0.076 | 3 |
| Predation*Species | 1.000 | 1.000 | 0.754 | 1.120 | 0.291 | 0.003 | 6 |
| Predation*Stream*Species | 5.000 | 5.000 | 11.176 | 3.320 | 0.006 | 0.042 | 4 |
| Error |  |  | 251.800 |  |  |  |  |
|  |  |  |  |  |  |  |  |
| c) Model for %C |  |  |  |  |  |  |  |
| Variable | Nparm | DF | Sum of Squares | F Ratio | P value | Partial η2 | Rank of effect |
| Stream | 5.000 | 5.000 | 252.149 | 4.590 | <.0001 | 0.058 | 2 |
| Species | 1.000 | 1.000 | 2.885 | 0.263 | 0.609 | 0.001 | 8 |
| Size | 1.000 | 1.000 | 4.956 | 0.451 | 0.502 | 0.001 | 7 |
| Predation | 1.000 | 1.000 | 57.279 | 5.214 | 0.023 | 0.014 | 5 |
| **Predation*Stream** | **5.000** | **5.000** | **294.384** | **5.359** | **<.0001** | **0.067** | **1** |
| Size*Species | 1.000 | 1.000 | 0.000 | 0.000 | 0.997 | 0.000 | 9 |
| Stream*Species | 5.000 | 5.000 | 90.508 | 1.648 | 0.147 | 0.022 | 4 |
| Predation*Species | 1.000 | 1.000 | 10.963 | 0.998 | 0.319 | 0.003 | 6 |
| Predation*Stream*Species | 5.000 | 5.000 | 118.591 | 2.159 | 0.058 | 0.028 | 3 |
| Error |  |  | 4108.700 |  |  |  |  |
|  |  |  |  |  |  |  |  |
