# Supplementary material for: Intraspecific variability modulates interspecific variability in animal organismal stoichiometry
Source: Ecol Evol. 2014 Mar 26;4(9):1505–15. doi: 10.1002/ece3.981 (PMC4063454; doi:10.1002/ece3.981)

Appendix 3. Averages (and standard errors) of elemental composition of *P. reticulata* and *R. hartii* collected from each stream, and from of the two predation communities.


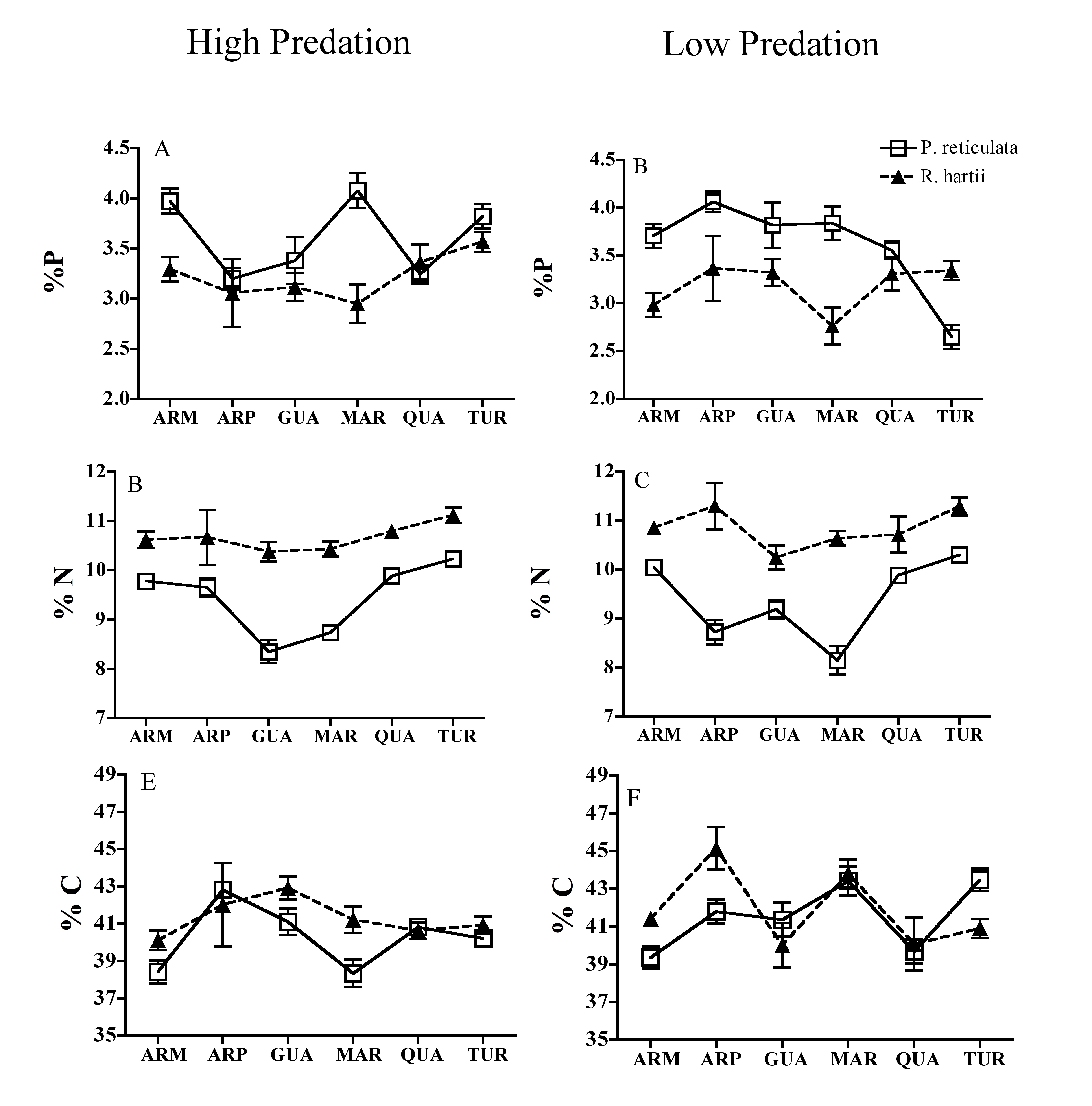

Supplement: Supplementary file 3 [file ece30004-1505-SD3.docx]
